# Editorial: Exosome-mediated intercellular communications: immune stimulation and suppression

**DOI:** 10.3389/fimmu.2024.1540484

**Published:** 2024-12-23

**Authors:** Nils Ludwig, Christopher W. Cutler, Ranya Elsayed

**Affiliations:** ^1^ Friedrich-Alexander University Erlangen-Nürnberg, Erlangen, Germany; ^2^ Periodontics Department, Dental College of Georgia, Augusta University, Augusta, GA, United States

**Keywords:** exosomes, immune stimulation, immune suppression, exosomal cargo, extracellular vesicles

In recent years, exosomes have emerged as crucial mediators of intercellular communication, fundamentally altering our understanding of biological signaling networks. These nano-sized extracellular vesicles (EVs), derived from multivesicular bodies and secreted by nearly all cell types, carry a diverse cargo, including proteins, lipids, RNA, and DNA. The Research Topic *“Exosome-Mediated Intercellular Communications: Immune Stimulation and Suppression”* aims to explore the multifaceted roles of exosomes in modulating immune responses, highlighting their potential in immunotherapy, diagnostics, and pathophysiology. This editorial provides an overview of the advances presented in this Research Topic, underscores the significance of these findings, and sets the stage for future research directions.

The Research Topic opens with a study exploring the diagnostic potential of exosomes in trauma. Schindler et al. identify novel EV biomarkers specific to traumatic brain injury (TBI), demonstrating how EVs with distinct protein profiles (e.g., CD13 and MOG) correlate with neurological outcomes. This work emphasizes the clinical value of EVs as minimally invasive tools for early diagnostics in polytrauma settings, illustrating the interplay between injury-specific exosome-mediated signaling and immune modulation.

Exosomes have recently been recognized as significant contributors to disease pathogenesis. In this context, the study conducted by Kobiela et al. highlights the role of keratinocyte-derived small extracellular vesicles (sEVs) in allergic skin inflammation, specifically regarding filaggrin insufficiency. Their study reveals that sEVs act as antigenic sources for CD1a-restricted T cells, promoting a type 2 immune bias that exacerbates atopic dermatitis. By linking lipidomic alterations in sEVs to immune dysregulation, the authors provide a mechanistic understanding of how exosome-mediated communication perpetuates skin inflammation, presenting opportunities for targeted interventions in allergic disorders.

Guo et al. delve into the immune-regulatory roles of exosomal microRNAs (miRNAs) in heart failure (HF). These miRNAs mediate the crosstalk between cardiac and immune cells, modulating processes like inflammation, fibrosis, and cardiac dysfunction. Highlighted as potential biomarkers and therapeutic agents, exosomal miRNAs could redefine diagnostic and therapeutic paradigms in HF, paving the way for more personalized approaches in cardiovascular medicine.

Exosomes have emerged as a promising candidate for various therapeutic applications across a range of diseases. Moreover, they possess the essential characteristics of an effective drug delivery system, enhancing their potential in medical treatments. In this context, a study by Wang et al. provided compelling evidence on the role of miR-146a-enriched fibroblast-like synoviocyte-derived exosomes (FLS-Exos) in alleviating osteoarthritis (OA). By targeting the Toll-like receptor 4/TRAF6/NF-κB signaling pathway, these exosomes reduced cartilage degradation and shifted synovial macrophages from the pro-inflammatory M1 phenotype to the anti-inflammatory M2 phenotype. This innovative approach highlights the potential of miR-146a as a therapeutic agent in OA, emphasizing the role of exosome-associated microRNAs in immune regulation and tissue repair.

Menay et al. explore the immunogenic potential of EVs derived from antigen-presenting cells (APCs) pulsed with foot-and-mouth disease virus (FMDV) antigens. These EVs carry viral proteins capable of stimulating both B and T cell responses, providing a new avenue for vaccine development. The ability of these vesicles to induce lymphocyte expansion, particularly in antigen-primed splenocytes, underscores their role as a platform for next-generation antiviral vaccines.

In another review, Mei et al. explore the therapeutic potential of mesenchymal stem cells (MSCs) as vehicles for drug delivery and immune modulation. The authors emphasize MSCs’ tumor tropism and homing ability, qualities that render them effective carriers for targeted therapies. They further discuss the promise of MSC derivatives, such as MSC-derived EVs and MSC cell membrane-coated nanoparticles, highlighting how these innovative strategies can enhance precision and efficacy in the treatment of inflammatory, fibrotic, and oncologic conditions. Despite the promise, challenges such as scalability and reproducibility must be addressed to ensure clinical translation.

The review by Xiu et al. sheds light on the crucial roles of bacterial membrane vesicles (MVs) in regulating bacterial physiology, environmental adaptation, and pathogenic processes. MVs are key players in interkingdom communication, influencing microbial community dynamics and host-pathogen interactions. The authors highlight their potential applications in developing novel antimicrobial strategies and vaccines, underscoring the importance of understanding bacterial MVs in both environmental and clinical contexts.

The Research Topic collectively demonstrates the multifaceted nature of exosomes, from their ability to stimulate immune activity to their role in suppressing inflammatory pathways. The therapeutic and diagnostic innovations explored here highlight the transformative potential of EV research, paving the way for novel applications in immunology and beyond.

We hope this Research Topic inspires future investigations into the intricate mechanisms of exosome-mediated communication and their implications for immune health and disease management. As the field continues to evolve, the convergence of clinical insights and translational innovations promises to redefine therapeutic paradigms.

For more details on these contributions, explore the articles in this Research Topic on the *Frontiers in Immunology* website.

